# Ultrasonography, X-ray and CT imaging findings of a giant pericardial lipoma: Imaging diagnosis and review of the literature

**DOI:** 10.3892/ol.2013.1668

**Published:** 2013-11-07

**Authors:** HAOHUI ZHU, MEIYUN WANG, DEGUANG FENG, YAN FENG, YING REN, JIYUN CHEN, YAO HE, JIANJUN YUAN

**Affiliations:** 1 Department of Ultrasound, Henan Provincial People’s Hospital, Zhengzhou, Henan 453000, P.R. China; 2 Department of Radiology, Henan Provincial People’s Hospital, Zhengzhou, Henan 453000, P.R. China; 3 Department of Cardiovascular Surgery, Henan Provincial People’s Hospital, Zhengzhou, Henan 453000, P.R. China; 4 Department of Neurology, Henan Provincial People’s Hospital, Zhengzhou, Henan 453000, P.R. China; 5 Department of Pathology, Henan Provincial People’s Hospital, Zhengzhou, Henan 453000, P.R. China

**Keywords:** pericardial lipoma, heart neoplasms, echocardiography, computed tomography, magnetic resonance imaging

## Abstract

Pericardial lipomas are rare and mostly asymptomatic tumors, which are usually detected incidentally during physical examination. The present study describes a case of giant pericardial lipoma that was diagnosed by surgical pathology. The study also describe the X-ray, magnetic resonance imaging, and the distinguish of the pericardial lipomas. The study also describes the ultrasonography, X-ray, computed tomography and magnetic resonance imaging findings of the tumor, and a review of the literature of cardiac lipoma, to help increase awareness of the tumor and selection of the correct imaging method for diagnosis.

## Introduction

Pericardial lipomas are rare and mostly asymptomatic tumors, which are usually detected incidentally during physical examination (1). The present study describes a case of a giant pericardial lipoma that was diagnosed by surgical pathology and presents the ultrasonography, X-ray, computed tomography (CT) and magnetic resonance imaging (MRI) imaging findings of the tumor in order to prevent a misdiagnosis. Written informed consent was obtained from the patient.

## Case report

A 45-year-old female suffered from post-exercise pressure in the chest for one year. The patient was diagnosed at Henan Provincial People’s Hospital (Zhengzhou, China). An electrocardiogram revealed a normal sinus rhythm without any remarkable abnormality.

**Figure 1:**
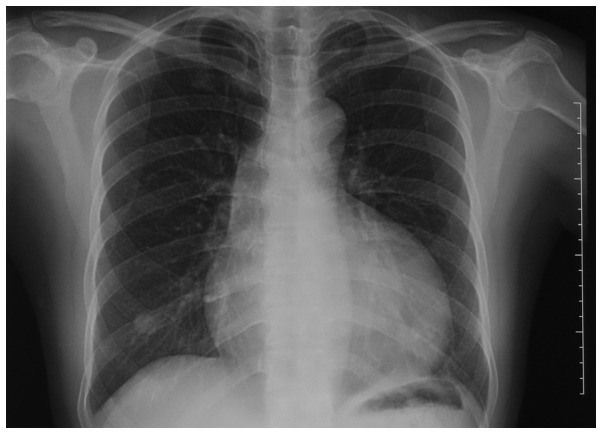
Chest X-ray reveals an enlargement of the cardiac silhouette. The cardiothoracic ratio is 64%.

**Figure 2:**
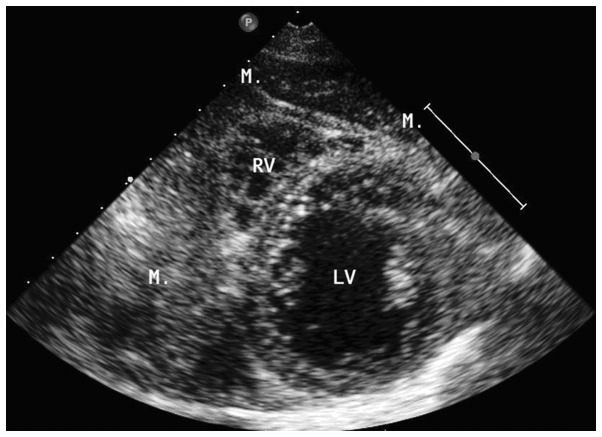
Parasternal short axis ultrasound showing a large mass abutting the left and right ventricle on the right, anterior and left epicardium surfaces. M, mass; RV, right ventricle; LV, left ventricle.

**Figure 3:**
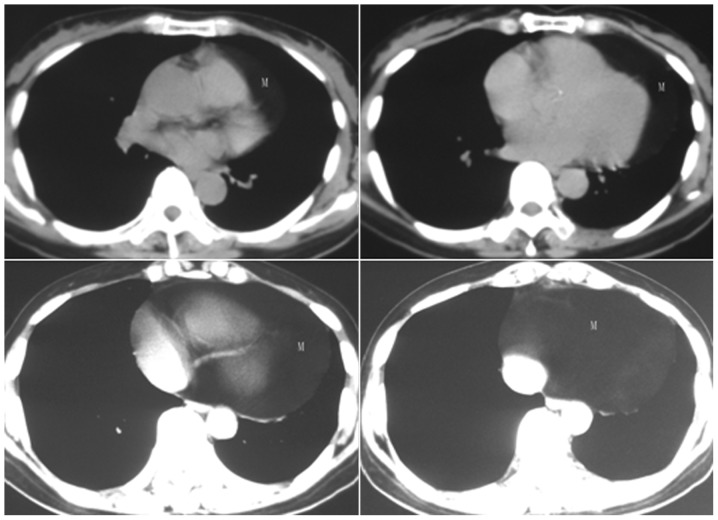
Multi-detector computed tomography plain scan images demonstrating a large lipoma in the anterior mediastinum surrounding the heart. The attenuation value of the mass was equal to that of subcutaneous adipose tissue. M, mass.

The chest X-ray (GE Feitian 6000 DR; GE, Buckinghamshire, UK) revealed a marked enlargement of the cardiac silhouette without any sign of pulmonary congestion and the cardiothoracic ratio was 64% (Fig. 1). A transthoracic echocardiogram (Philips IE33; Philips Healthcare, Eindhoven, Netherlands) demonstrated a huge echogenic mass compressing the left and right ventricle (Fig. 2). The size of the mass was 15.6×13.2×5.4 cm^3^ and the left ventricular ejection fraction was 66%. Multi-detector computed tomography (Philips Brilliance 16; Philips Healthcare) plain scan revealed a large mass along the right, anterior and left epicardial surface. The density of the mass was equal to that of subcutaneous adipose tissue (Fig. 3). 3D reconstruction and contrast CT was not performed in this patient, as the diagnosis of lipoma had been confirmed using echocardiography and a plain CT scan.

**Figure 4:**
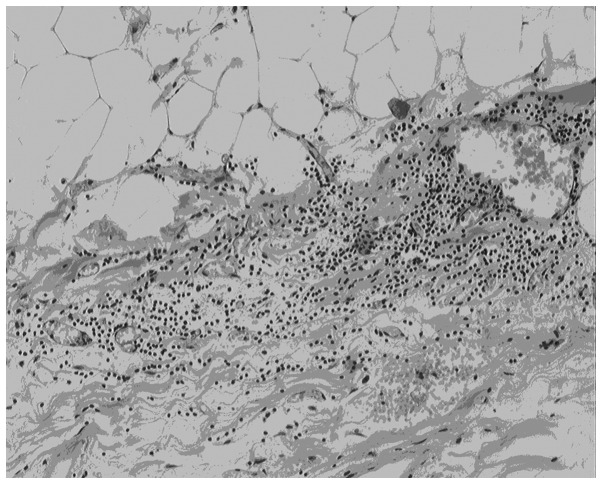
Histopathological examination of pericardial lipoma showing mature adipose tissue with inflammatory infiltration in the lipocytes and envelope (hematoxylin and eosin staining; magnification, 10×10).

The patient underwent surgery to remove the mass under the diagnosis of epicardial lipoma. During surgery, a large, yellow, soft, encapsulated tumor was identified at the surface of the heart. The tumor had a 1-cm pedicle that was connected to the anterior exterior epicardial surface of the left ventricle, with no invasion to the myocardium and pericardium. The mass was completely removed, weighed 1,550 g and was 16×14×4 cm^3^ in size. Histological examination revealed the nature of the tumor as mature adipose tissue with inflammatory infiltration in the lipocyte and envelope, confirming the diagnosis of lipoma (Fig. 4).

**Figure 5:**
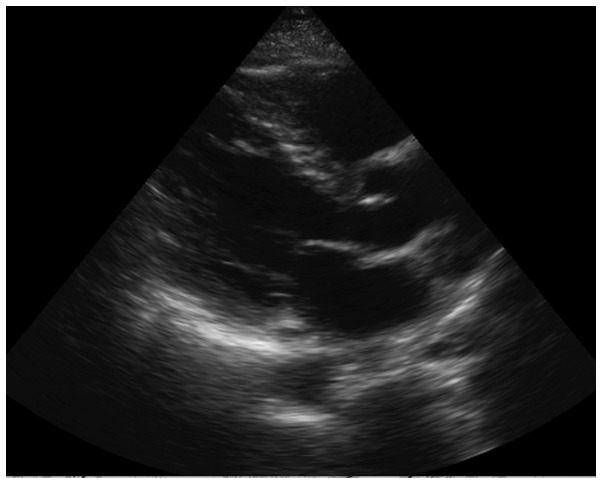
Echocardiography at the one-year follow-up shows no sign of recurrence.

The patient was discharged on the tenth postoperative day and remained asymptomatic in the following three months. The patient has demonstrated no sign of recurrence by echocardiography during every three-month follow-up for approximately one year following the surgery (Fig. 5).

## Discussion

Primary cardiac lipomas are rare benign tumors, which account for 10% of all primary cardiac tumors and 14% of benign cardiac tumors (2). The tumor may occur in females or males at any age. The majority of the patients are asymptomatic, while certain patients may suffer from discomfort in the chest, dyspnea, palpitation, syncope or sudden death, depending on the location of the tumor and the possible resultant compression or obstruction (3).

Generally, cardiac lipomas originate from the subendocardium, subpericardium or myocardium. A subendocardium lipoma may appear hemodynamically abnormal or display other symptoms depending on the location and the size. The tumor may cause valve regurgitation if the location is near the valve (4). Subpericardial lipoma is usually detected late in the clinic and may involve an extremely large mass that is either symptomless or causes symptoms including angina on exertion (by compressing the coronary arteries) and dyspnea (by tamponade). Intramyocardial lipoma may cause arrhythmias by interfering with the conduction system (2). According to the growth pattern, cardiac lipoma is classified into two types, invasive and non-invasive. The former usually infiltrates the adjacent tissue and is hard to be removed completely with a high recurrence rate. However, the latter has an envelope and is able to be resected completely with good prognosis (5).

In the diagnosis of cardiac lipoma, X-ray alone is not sufficient to reach an exact diagnosis, as it is easily misdiagnosed. The anatomical definitions, including the location, size, activity and hemodynamic consequences of the tumor may be assessed by transthoracic echocardiography, which is usually the initial examination for patients with a suspected cardiac mass. Sonography is able to identify an echogenic mass that is similar to the adipose tissue. When the adipose tissue causes liquefactive necrosis, it may appear as a low-level echo in the mass (6).

However, an accurate and reliable diagnosis must be obtained by CT (7) or MRI (8) with an ideal tissue resolution. The fatty composition of the tumor is easily identified with CT imaging and the complications of the tumor may be accurately depicted by reconstructed CT images (1). MRI scanning with multiple sequences has more advantages in the diagnosis of lipoma. The homogeneously fatty tumor with characteristic high-signal intensity on T1-weighted was effectively suppressed by the application of a fat saturation prepulse, which is highly specific for epicardial lipoma. In addition, complete morphological and functional assessment of cardiac tumors may also be performed by cardiac MRI (9). The patient in the present study was first diagnosed exactly by echocardiography and further examinations were performed using CT. However, a cardiac MRI examination was not performed for the present patient.

Lipoma should be distinguished from liposarcoma. CT or MRI are good tools for a differential diagnosis (10). As for liposarcoma, a plain CT scan usually shows mixed density (−80–40 hU) and calcification is occasionally observed. Contrast CT shows interior irregularity enhancement and MRI shows the funicular soft tissue in the fat tissue. Complete surgical excision of liposarcoma is the best choice, but the prognosis depends on the pathological type (11).

Pericardial lipoma should also be distinguished from mesothelioma of the pericardium, constrictive pericarditis, other secondary tumors of the pericardium, pericardial cysts and diaphragmatocele (4). Mesothelioma of the pericardium shows single or multiple tubercles and an irregular thickened pericardium (12), with or without hydropericardium (13). Constrictive pericarditis shows pericardium thickness, calcification and inferior vena cava broadening (14). A secondary tumor of the pericardium has the medical history of the primary tumor. When the echo level of pericardial lipoma is too low, it is similar to a pericardial cyst in the echocardiography, but CT and MRI are able to distinguish the two (15). When the content of diaphragmatocele is only adipose tissue, it is difficult to distinguish it from lipoma. The content of diaphragmatocele that is connected to the abdomen varies between the two (16).

In summary, although pericardiac lipoma is rare and seldom encountered, it is not difficult to obtain a correct diagnosis using the typical imaging findings. The present case aimed to aid the diagnosis of a cardiac tumor and propose the initial examination method of ultrasound, location, morphology, size, activity, internal echo, the association with the surrounding tissues and the hemodynamic changes. The method also has the advantage that the technique is cheap and convenient. CT and MRI have more ideal tissue resolution and a broader visual field than ultrasound, and are preferred in the imaging of associations with the mediastinum, lungs, diaphragm and other surrounding tissues. However, CT and MRI are more expensive than ultrasound and ultrasound is more convenient for a bedside examination.
